# Prevalence of binge drinking and associated behaviours among 3286 college students in France

**DOI:** 10.1186/s12889-016-2863-x

**Published:** 2016-02-23

**Authors:** Marie-Pierre Tavolacci, Eloïse Boerg, Laure Richard, Gilles Meyrignac, Pierre Dechelotte, Joël Ladner

**Affiliations:** Rouen University Hospital, Clinical Investigation Center 1404, 1 Rue de Germont, Rouen Cedex, 76031 France; Rouen University Hospital, IRIB, Inserm U1073 Rouen, France; Department of Nutrition, Rouen University Hospital, Rouen, France; Department of Preventive Medicine of University, Rouen University, Rouen, France; Department of Epidemiology and Health Promotion, Rouen University Hospital, Rouen, France

**Keywords:** Binge drinking, Alcohol, Tobacco, Cannabis, Sport, Stress, College students

## Abstract

**Background:**

Studies conducted on characteristics of binge drinking and associated behaviours in college student populations are scarce especially in France. Hence, it is important to identify risk factors for binge drinking at university, especially those which may be changed. The aim of this study was to assess the prevalence of binge drinking and associated behaviours across a large sample of college students in Upper Normandy (France).

**Methods:**

A cross sectional study was performed between November 2009 and February 2013 and data on socioeconomic characteristics and behavioural risk factors were collected: alcohol (consumption and misuse of alcohol, occasional and frequent binge drinking), tobacco, cannabis, cyberaddiction, stress and depression. An anonymous self-administered questionnaire was filled out by college student volunteers from Upper Normandy (France) either online or by paper questionnaire. Analyses were performed using multivariate logistic regression models.

**Results:**

A total of 3286 students were included. The mean (Standard Deviation (SD)) age of students was 20.8 years (SD = 2.1) with a male–female ratio of 0.60. The prevalence of binge drinking in the never, occasional and frequent categories was respectively 34.9 %, 51.3 %, and 13.8 %. The mean number of units of alcohol consumed per week (except BD episodes) was 0.78 for never, 3.7 for occasional and 10.5 for frequent binge drinkers (*p* < 0.0001). A positive relation was observed between frequent binge drinking and the following: male gender (AOR 4.77 95 % CI (3.43–6.63); *p* < 0.0001), living in rented accommodation AOR 1.70 95 % CI (1.21-2.40; *p* < 0.0001), attending business school AOR 4.72 95 % CI (2.76–8.08; *p* < 0.0001), regular practice of sport AOR 1.70 95 % CI (1.24–2.34; *p* = 0.001), smoking AOR 5.89 95 % CI (4.03–8.60; *p* < 0.0001), occasional cannabis use AOR 12.66 95 % CI (8.97–17.87;*p* < 0.0001), and alcohol abuse AOR 19.25 95 % CI (13.4–27.72; *p* < .0001). A negative association was observed between frequent binge drinking and grant holder status, living in couples, and stress.

**Conclusions:**

This study highlights the spread of binge drinking among college students and identifies student populations at risk: male gender, living in rented accommodation, regular practice of sport, and other risk behaviours such as use of tobacco, cannabis and alcohol. These behaviours increase with the frequency of binge drinking.

## Background

Binge drinking (BD) has been described as heavy alcohol use over a short period of time and is typically defined as four or five drinks in a row in women or men, respectively [1]. It is not easy to estimate the prevalence of BD in college students since published studies have reported different frequencies, ranging from a lifetime [2] to monthly [3] and weekly [4], in different populations, including adult [5], young adult [2], college students [4] and adolescents [6]. This dangerous pattern of alcohol consumption is highly prevalent among young adults and is a major public health concern in the USA [1] as well as most European countries [7, 8]. The prevalence of BD is highest among 18–24-year-olds [1, 9] and especially in college students [3]. The prevalence of BD among university students in France is about 30 %, 33 % – 60 %, and 11.0 % respectively when measured monthly [10], in the previous 2 weeks [4, 10, 11] and weekly. Alcohol misuse is highly prevalent among young people and especially college students [12]. Consequences of binge drinking at university include missed classes, lower grades, injuries, sexual assaults, overdosing, memory blackouts, changes in brain function, lingering cognitive deficits [13–16], and long term consequences such as morbidity and mortality [17, 18].

**Fig. 1:**
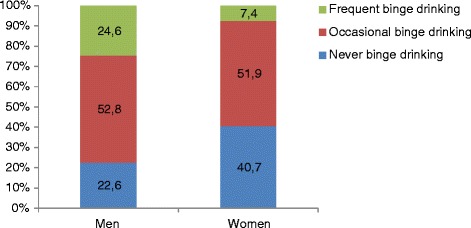
Frequency of consumption of binge drinking according to the gender of college students (France; 2010-2012) (*N*=3286)

Many studies on BD in college students involve medical students since future physicians are important opinion leaders and role models in terms of health related behaviours. Indeed, the drinking patterns of future physicians may affect how they treat patients with substance use disorders [19, 20]. In France, studies on BD at university focus mainly on students majoring in sport [21]. Variables that predict BD largely revolve around socialising (e.g., partying, and belonging to a social network of heavy drinkers) [22, 23]. Findings suggest that while some substance use represents a continuation of patterns initiated in high school, increased exposure and initiation of substance use frequently occur at university.

Adolescents and young adults who engage in BD are more likely to report other health risk behaviours such as cigarette smoking and/or cannabis consumption [24]. Furthermore, college students, especially female students, represent a stressed population with unhealthy risk behaviours regarding nutrition, alcohol and Internet. [25]. Despite efforts to promote health care, young adults continue to engage in high rates of risk behaviours [26]. There is growing recognition that post-secondary students should be a target population for health campaigns [27].

Studies conducted in college student populations on characteristics of BD and associated behaviours in college student populations are scarce especially in France [28]. Consequently, it is important to identify risk factors for BD at university, especially those which may be changed. The aim of this present study was to assess the prevalence of BD and associated behaviours across a large sample of college students in France.

## Methods

### Setting

Our health education programme focusses on students aged between 18 and 25 years in higher education in Upper Normandy (France). A dynamic partnership was formed with the regional multidisciplinary group “Ta Santé en 1 Clic” (TS1C): “Your health in one click”, involving partners from the health, higher education, and voluntary sectors as well as peer-students, all actively involved in the project design. The TS1C programme was based on two main actions: Health Forums on campus in Upper Normandy led by peer-students and a website specifically dedicated to students: www.tasanteenunclic.org.

### Design

We performed a cross sectional study between November 2009 and February 2013. This study design has been approved by the “Commission Nationale de l’Informatique et des Libertés” (The French Electronic Data Protection Authority). (Declaration number 1353247) and the Rouen University Hospital Institutional Review Board without mandatrory informed consent.

### Data collection

Between November 2009 and February 2013, students were invited to take part in the cross sectional study during health forum on campus, by email, or during their mandatory medical survey at the university medical department. The students filled out a confidential self-administered online on the TS1C website or by paper questionnaire. The questionnaire takes 20 min to complete. All questions in the online questionnaire were compulsory. Indeed, the online questionnaire was created with compulsory answers, meaning the student could not continue with the questionnaire if a previous question remained unanswered. Students could choose to answer all questions on the paper questionnaire, but this was not compulsory. Although the data was anonymous, a registration number was available for all students. This ensured there was no duplication.

### Socio-economic characteristics

The self-questionnaire included socio-economic characteristics: age, gender, grant holder status, student job status, accommodation status (at parents, rented accommodation, or on campus), and marital status.

### University curriculum

Five student groups were formed: 1) the mixed discipline university group, including faculties and schools specialising in literature, psychology, sciences, art and sport, 2) the health care group, including faculties and schools specialising in medicine, pharmacy, nursing, physiotherapy, midwifery and, radiology technologist studies, 3) the engineering group including engineering schools, 4) the business group, including business schools, and 5) the technology group, including different technical curricula of short duration. The academic year of study was also collected.

### Practice of sport

Students reported their practice of sport; leisure or competition, group or individual, and time spent per week. Weekly practice of sport was considered as regular.

### Assessment of alcohol use

#### Assessment of binge drinking

Binge drinking, defined as consumption of five or more alcoholic drinks (four or more for female students) on any one occasion, was classified as follows: more than twice a month as frequent, once a month or less as occasional, and total abstinence as never [1].

#### Assessment of consumption of alcohol except binge drinking

Alcohol consumption was classified as follows: on one or more occasion per week as frequent, on less than one occasion per week as occasional, and total abstinence as never. Data on the average number of units of alcohol consumed per occasion and the age at first episode of drunkeness were also collected.

#### Alcohol abuse problems

Alcohol abuse problems were assessed by ADOSPA [29] (Auto, Détente, Oubli, Seul, Probleme, Ami) test, the French version of the CRAFFT (Car, Relax, Alone, Forget, Family/Friends, Trouble) questionnaire [30]. This questionnaire is a mnemonic screen tool to identify alcohol abuse problems in young adults and contains 6 items that describe alcohol consumption for relaxation, alcohol consumption when alone, drinking alcohol and driving or riding with an alcohol drinking driver or rider, family or friends’ concern over alcohol consumption and, experiencing negative consequences of drinking alcohol. A score of 2 or more positive items usually indicates an alcoholic disorder.

### Assessment of other substance use

This study focussed mainly on the most common psychoactive substances used by students: tobacco, cannabis and alcohol. In the present study, regular smoking was defined as at least one cigarette per day, and occasional cannabis consumption was defined as at least one episode in the last 12 months according to standardized definition [31].

### Risk of cyberaddiction

Dr. Orman, author of the Internet Scale, assessed the tendency of becoming a net addict in a 9-item test (Valleur et al. 2002) [32]. Students with more than four positive answers were classified as beaing at risk of cyberaddiction.

### Stress and depression

A Perceived Stress Scale (PSS) was developed to measure the extent to which recent life status was appraised as stressful [33, 34]. The PSS is not a diagnostic instrument, so there were no cut-offs to determine stressed individuals and only comparisons between individuals were allowed. In our study, stress level was analysed by the median of perceived stress: students below the median were considered the least stressed and students above the median the most stressed. The 13-item version of the Beck Depression Inventory measures the intensity of depression, and has been validated for undergraduate students [35] and used in French [36]. The tool consists of 13 items in a 4-point scale (0–3), so that scores range from 0 to 39 (0 to 3: no depression; 4 to 7: low depression; 8 to 15: moderate depression and above 16: severe depression). Since few students were in the severe depression category, we grouped together the moderate and severe depression categories. This score was introduced in the questionnaire in December 2011. Data on depression were collected for 1689 of the 3286 students.

### Sleep duration

Students were asked to report their mean duration of sleep per night during the last week of the study. Insufficient sleep was determined as 7 h or less per night: epidemiological evidence indicates that a nightly sleep duration of 7–8 h per night is optimal, and is associated with overall good health status in adults [37].

### Statistical analysis

Students with missing data or outliers were excluded from the analysis. Thus, 3286 of the 3568 students included were analysed The Chi-square (χ^2^) test was used for comparisons of discrete data. Continuous variables were summarised by means and compared using the Student’s t-test. Factors with a p value below 0.20 were included in the multivariate analysis and a p value below 0.05 was considered to be significant. Logistic regression was performed to evaluate the independent determinants of occasional and frequent BD. Adjusted Odds Ratios (AOR) and their 95 % confidence intervals (CI) were calculated. Alcohol consumption was not included in the multivariate analysis because of interaction with BD. Statistical analysis was conducted using Xlstat® 2014.2.07 software package.

## Results

### Baseline characteristics of the study population

A total of 3286 college students with all data completed were included in the study between November 2009 and February 2013 (443 in 2009, 762 in 2010, 645 in 2011, and 1436 between 2012 and February 2013). The baseline characteristics are described in Table 1. The mean (Standard Deviation (SD)) age of students was 20.8 years (SD = 2.1) with a sex-ratio M:F of 0.60. 35.1 % of students were in mixed university group, 19.3 % in the health care group, 18.3 % in the engineering group, 14.3 % in the business group, and 13.0 % in the technology group. One third of students had a student job, one quarter had a grant and nearly half lived in rented accomodation.

**Table 1:** Baseline characteristics and frequencies of substance use of the 3286 French college students (France; 2010–2012)

	Never BD (*n* = 1146)	Occasional BD (*n* = 1686)	Frequent BD (*n* = 454)	Total (*n* = 3286)	*p*
Age years mean (SD)	20.5 (2.5)	20.4 (2.1)	20.6 (1.9)	20.5 (2.2)	0.80
Male gender (%)	24.2	37.6	64.4	36.6	<.0001
Student job holder (%)	20.9	20.5	21.5	20.8	0.89
Study grant holder (%)	41.7	31.3	24.6	34.0	<.0001
Students living (%)					<.0001
At parents	32.6	29.4	63.8	50.1	
In rented accommodation	42.2	52.0	23.1	29.7	
On campus	25.2	18.6	13.1	20.2	
Living in couples (%)	13.9	12.8	8.9	12.6	0.01
Curriculum (%)					<.0001
Technology	13.5	12.4	12.6	13.0	
Mixed university group	42.1	31.8	25.9	35.1	
Business School	6.2	14.9	34.8	14.3	
Engineering	17.8	20.1	15.1	18.3	
Health Care	20.4	20.8	11.3	19.3	
Academic year of study (%)					0.31
1	48.3	46.8	45.5	47.0	
2	16.4	15.8	16.7	16.3	
3	16.7	20.3	17.3	16.9	
>3	20.6	20.1	20.5	19.8	
Age at first episode of drunkeness mean (SD)	17.1 (1.8)	16.7 (1.7)	15.8 (1.7)	16.6 (1.6)	0.29
Regular practice of sport (%)	57.0	64.4	72.4	63.0	0.001
Length of sleep ≤7 h (%)	43.5	46.7	54.2	46.6	0.001
Depression^a^ (%)					0.003
No	51.3	53.4	60.3	53.2	
Low	23.9	28.9	19.9	26.0	
Moderate and severe	24.8	17.7	19.8	20.8	
Perceived Stress Scale (SD)	16.3 (7.4)	15.3 (7.2)	14.7 (7.0)	15.6 (7.3)	0.001
Smoker (%)	8.0	26.7	45.0	22.9	<.0001
Occasional cannabis user (%)	14.3	47.0	75.3	39.8	<.0001
Alcohol abuse problems (%)	6.6	29.0	66.5	26.6	<.0001
Risk of cyberaddiction (%)	22.7	25.9	30.5	25.4	0.008

BD: Binge drinking

Frequent BD: More than twice a month

Occasional BD: Once a month or less

Regular practice of sport: at least weekly

^a^:data collected in 1689 students

### Prevalence of binge drinking, alcohol consumption and alcohol abuse problems

The prevalence of never, occasional and frequent BD was respectively (1146/3286) 34.9 % (95 % CI = 33.2–36.6), (1686/3286) 51.3 % (95 % CI = 49.6–53.0), and (454/3286) 13.8 % (95 % CI = 12.7–15.0). Frequent BD was significantly more common in male students (24.6 %) than in female students (7.4 %); never BD was more common in female students (40.7 %) than in male students (22.6 %) (*p* < 0.0001) (Fig. 1).

The prevalence of never, occasional and frequent alcohol consumption (except episodes of BD) was respectively (450/3286) 13.7 %, (2251/3286) 68.5 % and (585/3286) 17.8 %. The mean number of units of alcohol consumed per week (except BD episodes) was 0.78 for non-binge drinkers, 3.7 for occasional binge drinkers and 10.5 for frequent binge drinkers (*p* < 0.0001).

Alcohol abuse problems (ADOSPA test) concerned 26.6 % of students (36.1 % male students vs 20.4 % female students (*p* < 0.0001) (Table 1).

### Characteristics of binge drinking and associated behaviours

Baseline characteristics according to frequency of BD (never, occasional and frequent) are described in Table 1. Concerning baseline characteristics in univariate analysis, BD differed according to gender, grant status, curriculum, living conditions, marital status, and sleep duration. Practice of sport was more common among occasional and frequent binge drinkers than among never binge drinkers. No difference was found in time per week dedicated to sport (global mean = 3.5 h/week SD = 3.0). Frequent BD was more common in college students playing team sports than individual sports (35.2 % vs 15.9 %; *p* < 0.0001), and there was no difference for never and occasional BD students. There were no significant differences related to age, student job status or year of study.

Logistic regression analyses compared occasional and frequent binge drinking students to never binge drinking students (Table 2). A positive relation was observed with male gender, business school attendance, practice of sport, regular smoking, occasional cannabis consumption especially with frequent BD (respectively AOR = 5.89 95 % CI = 4.03–8.60 and AOR = 12.66 95 % CI = 8.97–17.87), alcohol abuse problems and living in rented accommodation (only for frequent BD). Conversely, our results revealed a significant negative association between BD and stress, grant holder status, and living in couples (only with frequent BD). Binge drinking was not significantly related to sleep duration or risk of cyber addiction. Depression was not associated with BD for the sub group of 1689 students from whom these data were collected.

**Table 2:** Risk factors according to frequency of binge drinking among 3286 French college students (logistic regression) (France)

	Never BD (Ref)	Occasional BD		Frequent BD	
		AOR (95 % CI)	*p*	AOR (95 % CI)	*p*
Male gender	1	1.76 (1.42–2.18)	<.0001	4.77 (3.43–6.63)	<.0001
Study grant holder	1	0.78 (0.64–0.95)	0.01	0.66 (0.48–0.92)	0.01
Students living	1				
At parents		1		1	
In rented accommodation		1.14 (0.91–1.41)	0.24	1.70 (1.21–2.40)	0.002
On campus		0.81 (0.62–1.06)	0.13	0.81 (0.51–1.29)	0.37
Living in couples	1	0.80 (0.60–1.07)	0.13	0.44 (0.27–0.73)	0.02
Curriculum	1				
Technology		1		1	
Mixed university group		0.83 (0.62–1.12)	0.24	0.67 (0.42–1.08)	0.10
Business School		2.24 (1.50–3.36)	<.0001	4.72 (2.76–8.08)	<.0001
Engineering		1.50 (1.08–2.11)	0.02	1.35 (0.79–2.31)	0.27
Health Care		1.06 (0.76–1.48)	0.72	0.68 (0.40–1.17)	0.16
Regular practice of sport	1	1.24 (1.02–1.50)	0.03	1.70 (1.24–2.34)	0.001
Length of sleep ≤7 h	1	0.98 (0.90–1.18)	0.80	1.02 (0.76–1.37)	0.91
Perceived Stress Scale	1	0.75 (0.62–0.91)	0.004	0.72 (0.53–0.97)	0.04
Smoker	1	2.69 (2.00–3.63)	<.0001	5.89 (4.03–8.60)	<.0001
Occasional cannabis user	1	4.45 (3.52–5.61)	<.0001	12.66 (8.97–17.87)	<.0001
Alcohol abuse problems	1	4.39 (3.19–6.04)	<.0001	19.25 (13.4–27.72)	<.0001
Risk of cyberaddiction	1	1.06 (0.85–1.33)	0.59	1.27 (0.91–1.76)	0.16

*BD* Binge drinking

Frequent BD: More than twice BD a month

Occasional binge drinking: Once BD a month or less

## Discussion

To the best of our knowledge, this is the first study conducted on BD in France with a large sample of college students over a range of academic years (e.g., freshmen and senior students) and academic fields. Overall, we report binge drinking in two thirds of college students (almost 50 % occasional and 15 % frequent). In our study, we also highlighted the fact that almost one student in ten was at risk of frequent BD as well as alcohol consumption (except BD episodes).

### Socio demographic characteristics and BD

Male students had almost a 2-fold risk of occasional BD and a 5-fold risk of frequent BD compared to female students. This gender association but not the gradient is already known in college students and young adults [8, 38, 39]. Living in rented accommodation compared to living with parents and single status were also risk factors for frequent but not occasional BD. The review by Wicki et al. shows that students’ current living circumstances are clearly associated with alcohol consumption; those living in a “prototypical”, less controlled situation (e.g., living alone, with roommates, in student halls of residence or in areas with a high density of students) and without family obligations i.e., not living with their parents, their partner or their children were more likely to consume alcohol more frequently and in higher quantities [21]. In the present study, we have shown that study grant holder status protects from BD. These students probably do not have much money available to buy alcohol. Indeed Bartoli et al. previously reported that availabe money is a factor of BD: young adults with large amounts of money to spend at the weekend were more prone to BD [2].

### Binge drinking and associated behaviours

We found that practice of sport was associated with an almost 2-fold risk of frequent BD. Students playing team sports were especially involved in BD. Several studies have linked team sport athletes (e.g. football, rugby and soccer) with higher levels of alcohol consumption compared to individual sport athletes. This connection is likely due to the fact that drinking at a club is very much centred on team socialising, and further that such events often occur at a favourite bar or licensed clubhouse.

We have shown that BD is widely associated with cannabis and tobacco consumption regardless of cursus and year group. This association was previously demonstrated in young adults with quantity of alcohol but not with BD [2]. Association of tobacco and BD was also shown in a large US sample of college students [40]. The endangerment of college students and risk of addiction have been shown by positive ADOSPA test to increase with the frequency of BD as also clearly reported by White and Hingson [13]. These behaviours increase with the frequency of BD.

Negative association of stress with BD could be explained by use of BD as a mechanism for coping with stress. Hailing from cognitive-behavioural theories of addiction, the stress coping model of substance abuse suggests that people often consume alcohol as a coping response to stress [41]. Alcohol consumption allows for temporary relief from daily stressors, thereby reinforcing certain faulty coping strategies. According to Hasking et al., the relationship between avoidant coping and drinking behaviour is mediated by alcohol expectancies of increased confidence and tension reduction, which in turn are related to drinking motives [42].

Binge drinkers tend to have higher alcohol expectancies (beliefs an individual holds about the effects of alcohol) than social drinkers, and have a slightly lower level of drinking refusal [43]. Brief intervention alone should not be relied upon to address alcohol misuse in this population [44] and should be used in conjunction with effective environmental interventions such as restricting the physical availability and promotion of alcohol [45]. A recent randomised trial consisting in a web-based alcohol screening and brief intervention programme resulted in, at best, a small reduction in the amount consumed on a typical drinking occasion but not in other alcohol consumption and problem measures [46]. Ridoult et al. recently demonstrated implications for the use of Facebook to deliver positive messages on safe alcohol use to students, which may counter the negative messages regarding alcohol normally seen on Facebook [47]. New social networks such as Facebook could offer many advantages over delivery by more traditional social norms, providing an innovative method for tackling problem drinking at college.

Our study has certain limitations in as much as students were invited to participate in the study, and therefore the sample was not randomised. However the main characteristics of our sample are no different to those of a European study on 36000 French students [48]. Our study population was two thirds women and one third men (in the European study there were 63 % women and 37 % men). The mean age in our sample was 20.5 years compared to 21.2 years in the European study. The National Institute for Prevention and Education in Healthcare survey conducted in 2010 reported that 23.2 % of college students were smokers, a figure similar to ours [49]. So even if ours was a convenience sample, these characteristics do not seem to be different to those of other French university students and might be considered as representative of the student population However, since several curriculae and academic years of study were represented, the large sample might be considered as representative of the student population. In addition, this cross-sectional study was based on self-reported information provided by students which may represent some sort of bias, since participants may not be reliable in reporting their own behaviours. Heavier consumers might not have been present when the study was administered. Students might have under-reported their own substance use, as this measure was based on self-reporting. Self-reported substance use questionnaires have, however, been shown to be reliable for the substances studied [50, 51]. Cranford et al. reported that the main reasons given for not answering a questionnaire were students being too busy, or not interested, or forgetting to complete the survey [52]. The web-based socio-demographic and economic questionnaire provided information of similar-to-superior quality compared to the traditional paper version, with substantial logistic and cost advantages [53–55].

## Conclusion

Our study highlights the prevalence of binge drinking in college students and especially among male students. We also identify a target population for prevention and interventions: male students, living in rented accommodation, regular practice of sport, and other risk behaviours such as tobacco and cannabis use. Future interventions should involve all student year groups but could target specific curriculum.
